# Essential Oils from Wild Albanian *Lamiaceae*: GC-MS Profiling, Biological Activity, and Enhanced Delivery via Nanoencapsulation

**DOI:** 10.3390/molecules30163329

**Published:** 2025-08-09

**Authors:** Elton Basha, Erjon Mamoçi, Aniket Sharma, Entela Hodaj-Çeliku, Sanije Zejnelhoxha, Mădălina L. Medeleanu, Sonia A. Socaci, Bledar Bisha

**Affiliations:** 1 Department of Agri-Food Technology, Agricultural University of Tirana, 1025 Tirana, Albania; 2 Department of Food Science and Biotechnology, Agricultural University of Tirana, 1025 Tirana, Albania; mamocie@ubt.edu.al (E.M.); zejnelhoxhas@ubt.edu.al (S.Z.); 3 Department of Animal Science, University of Wyoming, Wyoming, WY 82071, USA; asharma7@uwyo.edu (A.S.); bbisha@uwyo.edu (B.B.); 4 Department of Chemistry, Agricultural University of Tirana, 1025 Tirana, Albania; ehodaj@ubt.edu.al; 5 Department of Food Science, Faculty of Food Science and Technology, University of Agricultural Sciences and Veterinary Medicine of Cluj-Napoca, 400372 Cluj-Napoca, Romaniasonia.socaci@usamvcluj.ro (S.A.S.)

**Keywords:** *Origanum vulgare* subsp. *hirtum*, *Thymbra capitata*, *Satureja montana*, antioxidant, antimicrobial, natural food preservatives

## Abstract

The growing demand for natural preservatives has driven interest in essential oils (EOs) from medicinal and aromatic plants. This study examines the potential of EOs from six wild populations of Albanian *Lamiaceae*, specifically *Origanum vulgare* subsp. *hirtum*, *Thymbra capitata*, and *Satureja montana* species, to be utilized for food conservation, among other possible uses. The EOs were extracted by hydrodistillation, and their chemical profiles were analyzed through GC-MS. DPPH and ABTS assays were performed to evaluate antioxidant activity. The antimicrobial efficacy of the oils was assessed using the broth microdilution method against six common foodborne pathogens: *Salmonella enterica* serovar Enteritidis, *Escherichia coli*, *Pseudomonas aeruginosa*, *Stenotrophomonas maltophilia*, *Micrococcus luteus*, and one fungus, *Candida albicans*. The most potent EOs in terms of yield and biological activity, resulting from *O. vulgare* subsp. *hirtum* and *T. capitata*, were encapsulated in oil-in-water emulsions, which were characterized for particle size and zeta potential. The results show that the populations of *O.vulgare* subsp. *hirtum* and *T. capitata* taken in the study belong to carvacrol chemotypes, and their EOs show strong antioxidant activity and are effective against all tested microorganisms. Nanoemulsions prepared with these EOs showed promising stability, indicating their potential as natural preservatives in food applications.

## 1. Introduction

The demand for natural preservatives that can replace artificial additives in food products is increasing due to a growing focus over food safety and shelf-life enhancement [1,2]. Although synthetic preservatives are effective, they often provoke health and toxicity concerns, leading customers to prefer more natural food additives. This has led to a renewed interest in plant-based natural preservatives, especially essential oils (EOs) [2]. EOs are complex mixtures of volatile organic compounds that are extracted from plants and possess a variety of bioactive qualities, among which are antioxidant, antimicrobial, and anticarcinogenic effects [3]. From the many plant families, the *Lamiaceae* family, which includes medicinal and aromatic plants (MAPs) like *Origanum*, *Thymbra*, and *Satureja* geniuses, has been found to contain considerable amounts of EOs with strong biological activity, which suggests that they could be used as natural food preservatives [4].

In this group, *Origanum vulgare* subsp. *hirtum* L., *Thymbra capitata* (L.) Cav., and *Satureja montana* L. are three MAPs used for culinary and medicinal purposes which are extensively found wild-grown in Albania and make up considerable export volumes for the country.

*Origanum vulgare* subs. *hirtum* (L.) is a perennial shrub with small green bracts and white flowers. It is very common in the spontaneous flora of Albania, Croatia, Greece, and Turkey [5]. In Albania, it can be found in the central part, but mostly it spontaneously flourishes in the south of the country [6]. Locally known as “White Oregano” (internationally referred as Greek Oregano) and is one of the two *Origanum vulgare* L. subspecies found in Albania, the other being “Red oregano” (*Origanum vulgare* L. subsp. *vulgare*). Compared to the *vulgare* subspecies, White Oregano contains higher amounts of essential oil [7,8], a reason for which it is widely used to produce commercial oregano EO, whereas its leaves are commonly applied as a spice in the Mediterranean culture [9]. The EO of *O. vulgare* subs. *hirtum* shows high concentrations of carvacrol and thymol as major constituents, accompanied by other metabolites in lower concentrations such as *p*-Cymene and γ-Terpinene [7].

*Thymbra capitata* (L.) Cav., also referred to as *Thymus capitatus* or *Satureja capitata* (L.) Cav. or *Coridothymus capitatus* (L.) Rchb.f., is a perennial and ornamental shrub endemic of the Mediterranean region [10]. It is known, especially for its medicinal uses, since ancient times [11]. In Albania, it is primarily located in the southern region, where it is often referred as “wild oregano”, owing to its aromatic similarity to the widely utilized White Oregano, likely due to the presence of the same principal constituents [12]. The extracts and EOs of *T. capitata* have been extensively analyzed for their phytochemical composition, revealing over 90 chemicals. The EO from *T. capitata* is mainly composed of phenolic monoterpenes such as carvacrol and thymol. Other compounds like *p*-cymene, γ-terpinene, β-caryophyllene, linalool, and borneol are significantly present. Usually, hydrodistillation is used to extract *T.capitata* EO, which reveals three chemotypes: carvacrol, thymol, and a combination of both [13].

*Satureja montana* (commonly known as winter savory) is also a perennial aromatic herb in the *Lamiaceae* family, native to the Mediterranean region and southern Europe. In Albania it is mostly found in mountainous areas even though it could be encountered from sea level. Same as for the two species described above, it is widely valued for its culinary and medicinal properties and is mostly used as a spice. Its EO, same as *O. vulgare* subsp. *hirtum* and *Thymbra capitata*, has the principal components carvacrol and thymol, which commonly occur with their chemical precursors *p*-cymene and γ-terpinene [12].

Carvacrol and thymol, two phenolic compounds possessing significant antibacterial and antioxidant properties, are prevalent in EOs derived from the *Lamiaceae* species, including *O. vulgare* subsp. *hirtum* L., *T. capitata*, and *S. montana* [14]. These compounds are suitable for food preservation applications, as they have been shown to inhibit lipid oxidation and microbiological proliferation [15].

One of the primary functions of EOs in food preservation is their antioxidant action, preventing the oxidation of lipids and other food constituents, which can lead to rancidity and nutrient degradation. Phenolic substances in EOs, which can act as hydrogen donors and neutralize free radicals, are mainly responsible for their antioxidant properties [3]. EOs from *Origanum*, *Thymbra*, and *Satureja* species have been demonstrated in multiple studies to possess antioxidant properties, effectively scavenging free radicals and protecting food from oxidative damage [14,16]. The robust antioxidant capacity of *T. capitata* and *O. vulgare* EOs has been evidenced by their notable DPPH and ABTS radical scavenging activities [17]. Likewise, *S. montana* essential oils have shown antioxidant properties comparable to synthetic antioxidants such as butylated hydroxyanisole (BHA) and butylated hydroxytoluene (BHT) [18]. The antioxidant qualities of the *Origanum* and *Thymbra* species are largely attributed to their high concentrations of carvacrol and thymol, with carvacrol being especially potent because of its capacity to donate hydrogen atoms in order to neutralize free radicals [19].

Additionally, EOs have a well-established antimicrobial profile and have been studied for their ability to stop the growth of bacteria and fungi. It is thought that EOs’ antimicrobial properties stem from their capacity to denature proteins, damage cell membranes, and obstruct the microbial metabolism [20]. Strong activity against a variety of foodborne pathogens, such as *S.* Enteritidis, *E. coli*, *P. aeruginosa*, and *C. albicans*, has been shown by EOs derived from the *Lamiaceae* species, especially *O. vulgare* and *T. capitata* [21,22]. In a similar fashion as for the antioxidant properties, the principal bioactive compounds in the *Origanum* and *Thymbra* EO responsible for their antibacterial effects are thymol and carvacrol. Thymol has been shown to possess bactericidal properties against both Gram-negative and Gram-positive bacteria, including *Salmonella enterica* and *Listeria monocytogenes* [23]. Thymol is a multipurpose substance for food preservation because it has antifungal as well as antibacterial qualities [24]. These EOs’ broad-spectrum antimicrobial activity has prompted research into using them as natural food preservatives because they can control the growth of pathogens and spoilage microorganisms that cause foodborne illnesses.

Even though EOs show significant potential as natural preservatives, their application in food systems is usually limited by their volatility, especially impacting the organoleptic profile, inadequate solubility, and low stability under various environmental conditions, such as temperature and light. Encapsulation has been proposed and explored to enhance the stability, bioavailability, and controlled release of EOs in food matrices in order to overcome these challenges. By adding active ingredients to nanoscale delivery systems like nanoemulsions, preservation of EOs from deterioration and their functional qualities are enhanced [25].

Oil-in-water nanoemulsions have emerged as an effective delivery system for essential oils, as they can encapsulate hydrophobic compounds like EOs, protecting them from oxidation and enhancing their stability during storage [26]. Since the smaller droplet sizes improve the dispersion of EOs in food products, fostering a better interaction with microorganisms and enhancing their antimicrobial and antioxidant effects, nanoemulsions not only offer stability but also the benefit of increased bioavailability [27]. *Origanum*, *Thymbra*, and *Satureja* EOs have been successfully encapsulated in nanoemulsions in a number of studies, indicating their potential as useful food preservation ingredients [28,29,30].

Because of its varied climate and flora, the territory of Albania is very rich in different MAPs. The country’s natural landscapes host extensive wild populations of *Origanum*, *Thymbra*, and *Satureja* species, traditionally utilized for culinary and medicinal applications. Albanian *Lamiaceae* species are an appealing subject for investigation owing to their potential in food preservation, with their biodiversity and optimal growing conditions [12]. Despite the growing number of studies on the biological activities of these plants, the literature still lacks information regarding the distinctive chemical profiles, antioxidant, and antibacterial properties of Albanian varieties of *Origanum vulgare* subsp. *hirtum*, *Thymbra capitata*, and *Satureja montana.*

This work aims to fill this knowledge gap by examining the yield and chemical composition of EOs from these species sourced from various regions of Albania, assessing their bioactive properties, and exploring the viability of their nanoencapsulation for enhanced stability and efficacy in food systems and potential use as natural food preservatives.

## 2. Results and Discussion

### 2.1. Yield of Extracted EOs

EO yield (Table 1), expressed as a percentage of dry plant material, is a critical parameter in assessing a plant’s industrial potential for EO production. *Origanum vulgare* subsp. *hirtum*, commonly known as Greek Oregano, demonstrated the highest EO yields among the studied species. The essential oil of *O. vulgare* subsp. *hirtum* had a pale-yellow color with a medium yield of 3.945% (*v*/*w*), a medium percentage compared to other studies of wild populations from Albania [8,31]. These results are consistent with previous studies confirming *O. vulgare* subsp. *hirtum* has superior EO yields compared to other subspecies, and the yield of the population taken in study, if cultivated, could be improved with appropriate agronomical practices [7,32,33]. High yields in this species are often associated with rich contents of carvacrol and thymol, compounds known for their strong antimicrobial activity [34]. The high yield makes this plant a promising candidate for cultivation and commercial applications in food preservation, pharmaceuticals, and aromatherapy.

*Thymbra capitata* produced moderate yields with a medium of 1.205% (*v*/*w*), and sample TC-M nearly doubled the yield of TC-L. EOs resulted in a pale-yellow color. The variability in EO percentage is commonly attributed to edaphoclimatic factors, as well as plant age and genotype [35]. The authors could not identify reports of the *Thymbra capitata* EO yield from Albania. Similar yields have been reported in North African regions, typically ranging from 0.5% to 2.0% [36]. While the yield is lower than *O. vulgare*, *T. capitata* oils are also rich in phenolic monoterpenes like thymol and carvacrol, providing potent antimicrobial and antioxidant effects that justify its use despite moderate yields [37].

*Satureja montana* samples displayed the lowest EO yields in this study, with a medium yield of 0.56% (*v*/*w*) where the EO had a pale-yellow color. SM-D had a considerably higher EO yield compared to SM-B, which would make it more promising for industrial applications. However, these values fall within the range reported in prior reports from Albania and other countries where EO yields for this species are typically between 0.25% and 1.69%, depending on location and genotype [12,38,39,40]. Despite the lower oil output, the *S. montana* EO is often high in components associated with strong biological activities [41]. Therefore, despite lower yields, the species might hold significance in specialized applications where quality outweighs quantity.

### 2.2. Quantitative and Qualitative Analysis of the EOs

GC-MS analysis of the *O. vulgare* subsp. *hirtum* EOs (Figures S1 and S2) identified, in total, 26 different components, accounting for 96.68% and 97.37% of the EOs of OV-P and OV-L samples, respectively. The most abundant compound was carvacrol, followed by γ-Terpinene, *p*-Cymene, and thymol accounting, on average, for 77.2%, 5.6%, 3.42% and 1.28%, respectively (Table 2). These results are in line with another report of *O. vulgare* subsp. *hirtum* from south Albania where carvacrol composed 79.8% of the EOs [31]. The EO of *O. vulgare* subsp. *hirtum* is clearly a carvacrol-type, which is consistent with previous chemotaxonomic classifications of Greek Oregano [42]. Carvacrol is well known for its strong antioxidant, antimicrobial, and also anti-inflammatory properties [43].

Both oils are very similar in chemical composition with minor differences, which are unlikely to significantly alter the overall bioactivity profile.

GC-MS analysis of the *T. capitata* EOs (Figures S3 and S4) identified, in total, 19 different components, accounting for 93.65% and 91.6% of the EOs of TC-M and TC-L samples, respectively. The most abundant compound was carvacrol, followed by *p*-Cymene, and γ-Terpinene, accounting, on average, for 75.25%, 4.48%, and 3.37%, respectively (Table 3). Consistency in major components indicates genetic stability despite the environmental variability of these two populations.

*T. capitata* samples clearly fit the carvacrol chemotype, similar to *Origanum* oils, and confirms its high bioactivity profile effective against Gram-positive and Gram-negative bacteria [44] and fungi [45]. The high carvacrol levels exceed many commercial standards, and in the case of TC-M, even the maximum level of 75%, set by The International Organization for Standardization (ISO 14717:2008) [46], places these oils among the most potent natural antimicrobial agents in the *Lamiaceae* family.

GC-MS analysis of the *S. montana* EOs (Figures S5 and S6) identified, in total, 47 different components, accounting for 98.33% and 80.83% of the EOs of SM-B and SM-D samples, respectively. The most abundant compound was thymol, followed by *p*-Cymene, carvacrol methyl ether, and γ-Terpinene, accounting, on average, for 40.65%, 10.35%, 5.35%, and 5.05%, respectively (Table 4). Thymol was markedly higher in the SM-B sample with almost double the concentration of SM-D.

*S. montana* oils display a thymol chemotype, particularly in SM-B. This population presents the highest thymol concentration compared to other reports from Albania [12,38,47], making it a valuable candidate for cultivation and industrial applications. Thymol is associated with potent antiseptic and antibacterial properties [48], which could be suitable for food preservation. The higher thymol content in SM-B indicates a stronger bioactivity potential compared to SM-D. However, SM-D shows greater chemical diversity, possibly due to genetic biodiversity from ecological and geographic variability [12].

**Figure 1:**
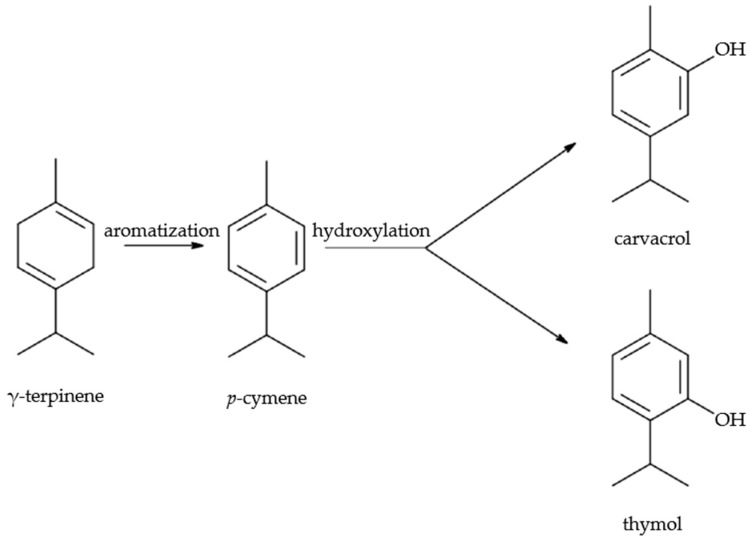
Four main active compounds of extracted essential oils, which in mean constitute 87.5%, 83.1%, and 57.9% of the total amount of the EOs from *O. vulgare* subsp. *hirtum*, *T. capitata*, and *S. montana* samples, respectively.

All three species contain biosynthetic precursors (γ-terpinene, *p*-cymene), showing enzymatic direction toward either carvacrol or thymol pathways (Figure 1) [49].

Carvacrol and thymol are isomeric oxygenated monoterpenes characterized by phenolic and hydroxyl structures that neutralize reactive oxygen species and free radicals through the transfer of electrons or hydrogen atoms [50]. Based on the carvacrol/thymol content, *O. vulgare* subsp. *hirtum* and *T. capitata* are stronger antioxidants/antimicrobials, while *S. montana* shows a slightly broader compositional complexity but lower potency. Oils rich in carvacrol (above 70%) are ideal for pharmaceutical and preservative uses. *S. montana* could appeal to niche markets that favor thymol-based formulations with milder olfactory characteristics.

### 2.3. Antioxidant Activity

**Figure 2:**
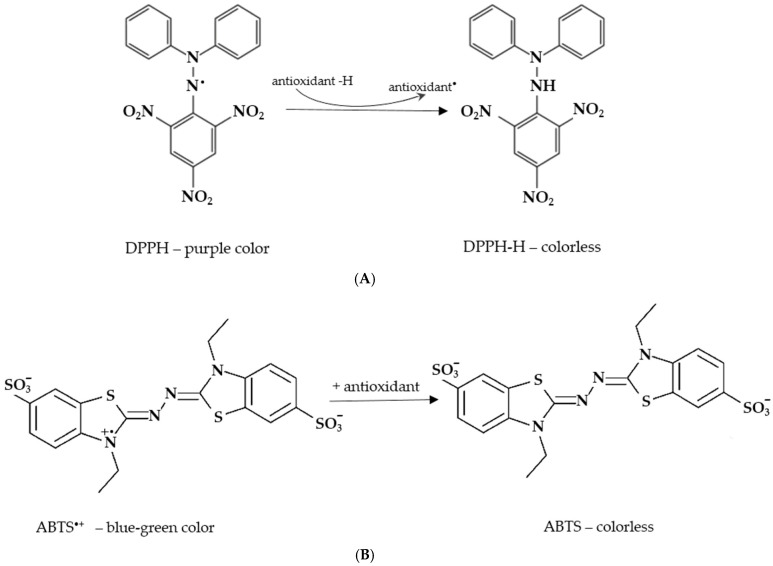
Free radicals used to assess the antioxidant activity of EOs. (**A**) DPPH (2,2-diphenyl-1-picrylhydrazyl) radical presenting a purple color (max. absorbance at 517 nm) and becoming colorless, usually after donation of a hydrogen by EO active compounds. (**B**) ABTS [2,2’-azinobis(3-ethylbenzothiazoline-6-sulfonic acid)] radical presenting a blue–green color (max. absorbance at 734 nm) that changes to colorless after EO active compounds donate a single electron or a hydrogen.

Antioxidant activity was evaluated through the capacity of the EOs to neutralize the DPPH and ABTS radicals (Figure 2A,B). The results, expressed as the EO concentration capable of neutralizing 50% of the free radical (IC_50_), are reported in Table 5. Lower IC_50_ values (μg/mL) indicate stronger antioxidant activity.

EOs of oregano samples OV-L and OV-P exhibited the lowest IC_50_ values, especially in the ABTS assay, indicating the strongest antioxidant capacity among all tested oils. This correlates strongly with their high carvacrol content (74.6–79.8%), known for its effective free radical scavenging and hydrogen donation ability due to its hydroxyl group and lipophilic character enhancing membrane interaction [51]. *T. capitata* EOs, also rich in carvacrol (72.8–77.7%), showed a similarly high antioxidant performance, although they were slightly less potent than the *O. vulgare*, possibly due to marginally lower carvacrol levels and total phenolics.

*S. montana* EOs demonstrated weaker antioxidant activity, especially SM-B, with the highest DPPH IC_50_ (1200 μg/mL). Despite having high thymol content (52.8% in SM-B; 28.5% in SM-D), the antioxidant potency was significantly lower than that of carvacrol-rich oils. Although thymol is also a phenolic compound, it is generally less effective than carvacrol in radical scavenging due to differences in the redox potential, steric hindrance from methyl groups [52], and electronic resonance stabilization of the phenoxyl radical.

The ABTS assay proved more sensitive across all oils, in accordance with what is generally reported in other works [53].

Oxidative degradation of food lipids leads to rancidity, loss of flavor, discoloration, and a decrease in nutritional value. EOs rich in carvacrol (e.g., *O. vulgare*, *T. capitata*) showed strong antioxidant activity [54], indicating their potential as natural alternatives to synthetic antioxidants like BHT and BHA. Their ability to scavenge both lipophilic (DPPH) and hydrophilic (ABTS) radicals broadens their applicability in emulsions, fats, meat products, and oils. While *S. montana* EOs were less effective as antioxidants, their aromatic and antimicrobial properties may still be valuable as sensory enhancers and microbial inhibitors, especially in combination with other preservatives.

### 2.4. Antimicrobial Activity

Table 6 shows the antimicrobial activity of the six EOs using the broth microdilution method in accordance with CLSI guidelines [55].

MICs were determined and ranged from 0.15 to 2.5 mg/mL. *C. albicans* and *S. maltophilia* were the most susceptible organisms overall, while *P. aeruginosa* was the most resistant. *P. aeruginosa* is known for its resistance mechanisms, including low outer membrane permeability and active efflux systems, which limit the efficacy of hydrophobic substances like EOs [56].

*O. vulgare* subsp. *hirtum* EOs showed a strong activity (MIC ≤ 0.625 mg/mL) against *E. coli*, *M. luteus*, *S. maltophilia*, and *C. albicans*, likely due to high carvacrol content (74.6–79.8%), which is known to disrupt bacterial membranes and induce the leakage of cytoplasmic contents [22]. Slight differences in MIC values between OV-L and OV-P EOs may reflect minor differences in the thymol content or minor components.

In the *T. capitata* sample, TC-M showed broad-spectrum activity with very low MICs against *C. albicans* (0.156 mg/mL) and *E. coli* (0.312 mg/mL). TC-L had no inhibitory effect on *P. aeruginosa*, indicating the possible influence of a lower carvacrol content (4.9% less) and other minor constituents. Still, their antimicrobial potency is explained through their high carvacrol content (72.8–77.7%).

*S. montana* samples demonstrated the weakest antimicrobial activity, with the highest MIC levels on all tested strains compared to the other two species taken in the study. Lower efficacy is consistent with a lower carvacrol content and higher relative content of thymol and monoterpenes, with weaker antimicrobial effects (e.g., borneol, *p*-cymene).

Carvacrol is generally reported to have stronger antimicrobial activity than thymol, although both are potent and structurally similar phenolic compounds [57].

The variation in antimicrobial activity between the EOs of the same species can be attributed to small differences in chemical composition where the interaction among major and minor compounds could have synergistic or antagonistic interactions which explain the differences in MICs [58].

To our knowledge, this is the first report on the activity of the EO from *S. montana* on the multidrug-resistant *S. maltophilia* bacteria with MIC doses of 1.25 and 0.625 mg/mL, SM-B and SM-D, respectively.

To contextualize the observed MIC values of EOs, it is useful to compare them with conventional antibiotics. For instance, ciprofloxacin, a widely used fluoroquinolone, typically exhibits MICs of 0.015–0.25 µg/mL against *Escherichia coli and Pseudomonas aeruginosa* [59]. These values are significantly lower by mass than those observed in the studied EOs (typically 0.156–2.5 mg/mL), yet this comparison underscores the difference in mechanisms of action and possible synergistic use of antibiotics combined with EO compounds [60,61,62,63,64,65]. The use of natural products as antibiotic adjuvants to enhance the efficacy and mitigate resistance is increasingly recognized as a promising strategy. For example, in the study conducted by Ghan C., et al. (2025) [66], synergistic antimicrobial combinations of carvacrol significantly reduced the required antibiotic dose by 4- to 16-fold. This strategy significantly lowers the risk of antimicrobic resistance, not only because the antibiotic dose is significantly lowered, but also because antibiotics act by a specific mechanism of action, while EOs—particularly those rich in carvacrol and thymol—exert multi-targeted effects, including membrane destabilization, the disruption of proton gradients, disrupting the ATP metabolism, and interference with efflux pump activity [66,67]. This results in a promising strategy against multidrug-resistant pathogens which nowadays are a global concern [68]. On the other hand, in the context of food preservation, natural preservatives containing carvacrol and thymol have been shown to also be effective against biofilms [69,70,71,72], which are problematic even for the commonly used synthetic preservatives in the food industry. Although EOs cannot directly replace conventional antibiotics, their broad-spectrum antibacterial properties, synergistic potential with antibiotics, and multi-target mechanisms render them significant allies in the fight against bacteria.

On the other hand, EOs are generally recognized as safe (GRAS) when used at appropriate concentrations. In the context of targeting spoilage and pathogenic microorganisms, EOs from *O. vulgare* and *T. capitata*, due to their low MICs and broad-spectrum efficacy, are suitable for preserving perishable foods such as minced meats and sausages, ready-to-eat salads or dips, dairy products, etc. [73,74]. The strong aroma and flavor of carvacrol-rich oils may require dose optimization in combination with other hurdles (e.g., refrigeration, mild heat, or acids) [75] or better incorporation in delivery systems, such as edible films and coatings [76] or nanoemulsions [77] for an improved dispersion in aqueous food systems to enhance efficacy and minimize sensory impacts.

As encapsulation and nanoemulsion technologies can improve stability, solubility, and controlled release, minimizing the flavor impact while preserving efficacy, the most promising EOs were tested for incorporation in a nanoemulsion delivery system.

### 2.5. EOs Nanoemulsions

To be ideal candidates for incorporation into nanoemulsions, EOs should meet the following: high yield—to ensure feasibility and cost-effectiveness; strong antioxidant activity—to prevent oxidative deterioration; and potent antimicrobial activity—to inhibit or eliminate foodborne pathogens, chemical stability and compatibility with emulsifiers and food matrices.

From the plant species taken in our study, *O. vulgare* subps. *hirtum* and *T. capitata* provided the most prominent results, respectively represented by EOs of samples OV-L and TC-M which performed better. Specifically, OV-L had a slightly higher yield and slightly stronger antioxidant and antimicrobial activity compared to OV-P. TC-M, in general, performed better than the TC-L sample (Table 7). Nevertheless, high EO yields in *O. vulgare* subps. *hirtum* (3.8–4.1%) ensure scalability and economic viability. Although the yield is lower in *T. capitata* samples, its chemical richness in carvacrol, potent antifungal and antibacterial activity, and strong antioxidant action justify its use in targeted nanoemulsion formulations.

Nanoemulsions were formed using water and sunflower oil, which are all recognized as GRAS, and Tween 80, a permitted food additive by the Food and Drug Administration of the USA. Due to its safety profile, Tween 80 (T80), sometimes referred to as Polysorbate 80, is widely used and has been approved by the European Food Safety Authority (EFSA) for use in food items [78].

Results from the DLS (Dynamic Light Scattering) of nanoemulsions incorporating OV-L and TC-M EOs, for the particle size, polydispersity index (PDI), and Z-potential, which are key parameters influencing the stability, bioavailability, and functionality of EO nanoemulsions in food systems, are shown in Table 8.

According to the literature, emulsion droplets with size < 200 nm are ideal for improved optical clarity, enhanced bioavailability and penetration into microbial membranes, and greater colloidal stability due to a reduced gravitational separation [79]. The OV-L EO’s nanoemulsion particle size was in the optimal nano-range (<200 nm) with a mean size of 132.5 nm, which indicates well-dispersed and finely emulsified droplets; meanwhile, the TC-M EO’s nanoemulsion was in the nano-range, even though it was at the upper end limit.

The OV-L nanoemulsion mean PDI of 0.152 categorizes it into the moderately monodisperse range (0.1–0.3), which is typically considered suitable for nanoemulsion systems [80]. The significant variability between replicates suggests that additional process optimization may be necessary. On the other hand, the TC-M PDI value classifies it as very monodisperse (PDI < 0.1), indicating good droplet homogeneity and system stability. The standard deviation of 0.096 is relatively moderate and can likely be attributed to inherent batch variability rather than instability. Moreover, research on EO nanoemulsions stabilized with Tween 80 demonstrates that low polydispersity index values are associated with an extended shelf life and reduced phase separation [81].

Zeta potential may act as a partial indicator of the physical stability of the emulsion created. The higher the absolute value of the zeta potential, the more stable an emulsion is. To ensure the formation of a robust energy barrier against the coalescence of dispersed droplets, it has been advised to attain high-absolute zeta potential values (exceeding ±30 mV) in most prepared emulsions [82]. The formed nanoemulsions showed low zeta potential values, indicating a possible low stability. Nevertheless, analyzing the zeta potential for a nanoemulsion stabilized with Tween 80 is not always essential for understanding stability—and its interpretive value is limited, because Tween 80 provides a primarily steric, rather than electrostatic, stabilization by forming a hydrated, bulky layer around droplets, preventing coalescence through physical hindrance rather than electrostatic repulsion. For example, Tan, T.B., et al. [83] reported that no increase in particle size was seen for the Tween 80-stabilized nanodispersion, even when zeta potentials were brought near 0 mV, due to the non-ionic characteristics of the Tween 80 emulsifier, which impart stability via steric hindrance.

In summary, it can be deduced that both nanoemulsion formulations were good and need to be further tested for stability and efficacy, especially in specific food applications. The OV-L EO nanoemulsion offers the best size characteristics for transparent or colloidally stable food systems (e.g., beverages, edible films), though stabilization can be further optimized. TC-M demonstrates superior formulation, which is essential for prolonged commercial application.

## 3. Materials and Methods

### 3.1. Plant Material

Six plants, two of each concerned species, *O. vulgare* subsp. *hirtum*, *T. capitata* and *S. montana*, from wild-growing populations, were collected in August 2023, as they are known to accumulate higher amounts of essential oil when exposed to high radiation and temperature [35,84,85].

Information on the collection places of these populations is shown in Table 9.

The species identification for each population was carried out by the Genetics and Plant Breeding Laboratory, Agricultural University of Tirana, Albania, and the herbarium specimen vouchers were deposited in the same laboratory. The plant materials were dried in a well-ventilated, shaded area at room temperature, about 25 °C, and relative humidity around 50%. Once dried, stems were separated from leaves and flowers.

### 3.2. EOs Extraction

The EOs were extracted by hydrodistillation using a Clevenger apparatus [86]. One hundred grams of dried plant material were minced finely and put into a one-liter flask with 500 mL of distilled water. Distillation went on for three hours at a rate of three milliliters per minute. The oil yield was calculated as a percentage of volume by weight (% *v*/*w*) relative to the dry weight of the plant material. The obtained EOs were stored at 4 °C prior to analysis.

### 3.3. Reagents and Microbial Strains

Antioxidant radical screening reagents 2,2-diphenyl-1-picrylhydrazyl (DPPH) and 2,2’-azinobis(3-ethylbenzothiazoline-6-sulfonic acid) (ABTS)] were purchased from Alfa Aesar (Haverhill, MA, USA). Methanol was secured from VWR International (Fontenay-sous-Bois, France) and ethanol from Merk KGaA (Darmstadt, Germany).

The bacterial American-type culture collection strains *S. enterica* serovar Enteritidis (ATCC:49223), *E. coli* (ATCC:10535), *P. aeruginosa* (ATCC:9027), *S. maltophilia* (ATCC:13637), and *M. luteus* (ATCC:10240), along with one fungal isolate *C. albicans* (ATCC:10231), were procured from Microbiologics, Inc. (Saint Cloud, MN, USA); 96-well plates were secured from Corning Inc. (Corning, NY, USA). Blood agar medium and Mueller Hinton Broth were procured from Remel Inc. (Waltham, MA, USA), 0.5 Polymer McFarland Standard from Thermo Fisher Scientific (Waltham, MA, USA), and Dimethylsulfoxide (DMSO) from Sigma-Aldrich (Saint Louis, MO, USA).

### 3.4. Gas Chromatography–Mass Spectrometry

Gas Chromatography–Mass Spectrometry EO analyses were performed on a Shimadzu GC-2010-GCMSQP2010 (Kyoto, Japan) system operating at 70 eV. The temperature program was from 60 °C to 250 °C, at a rate of 5 °C/min. Helium was used as a carrier gas at a flow rate of 1.0 mL/min. The injection volume of each sample was 1 μL. Retention times for all compounds were determined according to Van den Dool and Kratz, 1963 [87], using n-alkanes as standards. The identification of the components was based on a comparison of their mass spectra with those of NIST21 and NIST107 [88], and by the comparison of their retention indices with the literature data, Adams, 2007 [89]. Component-relative concentrations were calculated based on GC peak areas without using correction factors. EOs were often subjected to co-chromatography with authentic compounds procured by Fluka, Sigma (Buchs, Switzerland).

### 3.5. Free Radical Scavenging Activity

The free radical scavenging activity of the essential oils was measured in vitro using DPPH and ABTS assays according to Brand-Williams et al., (1995) [90], and to Re et al., (1998) [91], respectively. The stock solution for the DPPH assay was prepared by dissolving 24 mg of DPPH in 100 mL of methanol and storing it at 20 °C. The working solution was obtained by diluting the DPPH stock solution with methanol to achieve an absorbance of about 0.98 ± 0.02 at 517 nm using a spectrophotometer (Biochrom Ltd. Libra S22—Cambridge, UK). The stock solution for the ABTS assay was prepared by dissolving ABTS in water at a concentration of 7 mM. The ABTS radical cation (ABTS^•+^) was generated by mixing the ABTS stock solution with potassium persulfate at a final concentration of 2.45 mM, followed by incubation in the dark at room temperature for 12–16 h. The working solution was then prepared by diluting the ABTS stock with ethanol until the absorbance reached approximately 0.70 ± 0.02 at 734 nm, as determined using the spectrophotometer.

Three mL aliquots of each solution were combined with 77 μL of the sample at 6 concentrations (50, 100, 200, 5000, 1000, and 2000 μg/mL) then thoroughly mixed. For the DPPH assay, mixtures were incubated in the dark at room temperature for 30 min, then the absorbance was measured at 517 nm. For the ABTS assay, the absorbance was recorded at 734 nm after 5–6 min of reaction in similar conditions. The controls were prepared as above but without essential oil. The activity was evaluated based on the percentage of the DPPH and ABTS radicals removed as the following equation:

Inhibition of the free radical (%) = [(A_control_ − A_sample_)/A_control_] × 100

where A_control_ is the absorbance of the control which contains all the reaction components, except the test sample, and A_sample_ is the absorbance of the test compound.

The result was calculated as the concentration of essential oil that inhibits 50% of the free radical (Inhibition Concentration—IC_50_).

### 3.6. Evaluation of Antimicrobial Activity by Microdilution Broth Method

The antimicrobial activities of the EOs were evaluated against a panel of clinical and foodborne pathogens using standard American Type Culture Collection strains (ATCC) and five bacteria, along with one fungal isolate. Stock cultures were maintained at 4 °C and subcultured immediately before use. Prior to EO treatment, bacterial strains were incubated at 37 °C and the fungal isolate at 28 °C, each for 18–20 h on blood agar, ensuring cultures were in optimal growth phase.

The MIC (Minimum Inhibitory Concentration) of each EO was determined using a broth microdilution method in 96-well plates following the Clinical & Laboratory Standards Institute (CLSI) protocols [54]. In brief, bacterial suspensions were adjusted to a final concentration of 10^5^ CFU/mL cells standardized by 0.5 McFarland in Muller Hinton Broth (MHB) media. EO stock solutions were prepared at 100 mg/mL by dissolving them in DMSO. From this stock, a working solution of 5 mg/mL was subsequently diluted in MHB media, ensuring the final DMSO concentration is less than 5%. Serial 2-fold dilutions were performed to achieve EO concentrations ranging from 5.0 to 0.0097 mg/mL in the microplate wells. Finally, 100 µL of the bacterial suspension was added to each well. The plate setup included the 11th column as the media control (negative control) and the 12th column containing bacteria and media (positive control). Additionally, rows D and E were designated for solvent controls, with row D with only the EO (solvent control) and row E the DMSO (concentration used to dissolve EO) and bacteria served as the DMSO control. The plates were incubated at 37 °C for 24 h. MIC was determined using Tecan i-control 2.0 software (Infinite M Plex TECAN—Männedorf, Switzerland) to measure the Optical Density (OD) at 600 nm compared with the positive control. Everything was kept constant for determining antifungal activity except incubation, which was performed for 45–48 h, with the OD determined at 530 nm. MIC was determined as the lowest concentration of EO that inhibited the visible growth of the tested microorganism [92].

### 3.7. Encapsulation of EOs

The oregano and thyme EO nanoemulsions were prepared by using the method previously described by Bodea, Cătunescu, Palop, Fernandez, and Garre (2023) [93], with some modifications. Specifically, a total oil phase was formed with 2 mL of oregano EO and 1.35 mL of sunflower oil as the carrier oil. A coarse emulsion was first produced by mixing the oil phase and 2.5 mL Tween 80, followed by combining it with deionized water added dropwise under continuous stirring at room temperature. The emulsion formed after 4.15 mL of water was added. For the thyme EO nanoemulsion, the constituents were in slightly different concentrations: 1 mL of EO, 1.0125 mL of sunflower oil, and 1.875 mL of Tween 80. The coarse emulsion was homogenized by ultrasonification (USF) for 15 min at 100% amplitude to obtain the functionalized nanoemulsion. The maximum temperature of the samples during sonication was kept at 25 °C. Nanoemulsions were immediately stored in refrigeration and analyzed after two weeks.

The average droplet size and zeta potential were determined by dynamic light scattering (DLS) (Zetasizer Nano, Malvern, UK).

### 3.8. Statistical Analysis

All experiments were performed in triplicate and the standard deviation calculated. The IC_50_ value was determined by simple linear regression using the percentage inhibition data over the linear dose range. The standard deviation of the IC_50_ was calculated by error propagation, based on the standard errors of the slope and intercept. The analyses were performed with GraphPad Prism software 10.5.0.

## 4. Conclusions

From six wild populations of Albanian *Lamiaceae*, two specimens each of *O. vulgare* subsp. *hirtum* and *T. capitata* exhibited the carvacrol chemotype, whereas samples of *S. montana* showed the thymol chemotype. Oregano samples showed the highest essential oil yields, rendering them attractive for prospective industrial production.

The extracted essential oils demonstrated potent antioxidant properties and were effective against six widespread pathogens, encompassing both Gram-negative and Gram-positive bacteria, as well as a fungus, thus confirming their broad-spectrum antibacterial activity.

The most promising essential oils, with regard to yield and biological activity, derived from *O. vulgare* subsp. *hirtum* and *T. capitata*, were effectively encapsulated in nanoemulsions that could have a wide array of applications, including incorporation into food products due to their composition in permissible substances. Nevertheless, the stability and efficacy of these nanoemulsions need to be further tested, especially in real-food conditions. Consequently, perhaps alongside other obstacles, they may serve as effective alternatives to synthetic preservatives which are increasingly seen as undesirable.

## Figures and Tables

**Table 1:** Yield of extracted EOs.

Nr.	Species	Plant Code	Yield of Aerial Parts (% *)	Yield (% *) of Leaves and Flowers Without Stems
1.	*Origanum* *vulgare* subsp. *hirtum*	OV-L	4.06	6.19
2.		OV-P	3.83	6.11
3.	*Thymbra* *capitata*	TC-M	1.66	3.00
4.		TC-L	0.75	2.30
5.	*Satureja* *montana*	SM-B	0.39	0.80
6.		SM-D	0.73	0.79

* mL of EO per 100 g of dried plant material.

**Table 2:** Composition of the essential oils of *O. vulgare* subsp. *hirtum*.

Compounds ^a^	AI ^b^	OV-P (% ^c^)	OV-L (% ^c^)	ID ^d^
α-Thujene	924	1.16	0.97	AI, MS
α-Pinene	931	0.95	0.75	AI, MS, Co-GC
Camphene	945	0.54	0.66	AI, MS
1-Octen-3-ol	983	0.6	0.6	AI, MS
β-Myrcene	990	1.5	1.5	AI, MS, Co-GC
δ-2-Carene	1003	0.2	0.14	AI, MS
α-Phellandrene	1005	0.2	0.2	AI, MS
α-Terpinene	1016	0.98	1.03	AI, MS
*p*-Cymene	1025	3.8	3.04	AI, MS, Co-GC
Sylvestrene	1029	0.3	0.26	AI, MS
Eucalyptol	1030	0.36	nd	AI, MS
*trans*-Ocimene	1038	0.1	0.08	AI, MS
*cis*-Ocimene	1050	0.1	0.06	AI, MS
γ-Terpinene	1059	5.8	5.4	AI, MS, Co-GC
*trans*-Sabinenehydrate	1070	0.46	0.34	AI, MS
Terpinolene	1085	0.1	0.07	AI, MS
Linalool	1102	0.1	nd	AI, MS, Co-GC
α-Thujone	1104	0.1	0.07	AI, MS
Camphor	1143	0.15	0.13	AI, MS
Terpinen-4-ol	1181	0.15	0.09	AI, MS, Co-GC
Thymol	1294	2.32	0.23	AI, MS, Co-GC
Carvacrol	1304	74.6	79.8	AI, MS
α-Ylangene	1371	0.1	0.05	AI, MS
β-Caryophyllene	1417	1.1	1.2	AI, MS, Co-GC
α-Humulene	1454	0.16	0.2	AI, MS, Co-GC
Caryophyllene oxide	1582	0.75	0.5	AI, MS, Co-GC
Total		96.68	97.37	

^a^ Compounds listed in order of elution from an HP-5 MS capillary column; ^b^ AI: arithmetic indices as determined on an HP-5 MS capillary column using a homologous series of n-alkanes (C9-C23); ^c^ percentage (*w*/*w*) of the identified compound in the essential oil; and ^d^ identification method: AI = arithmetic index, MS = mass spectrum, Co-GC = co-injection with authentic compound, and nd = not detected.

**Table 3:** Composition of the essential oils of *T. capitata*.

Compounds ^a^	AI ^b^	TC-M (% ^c^)	TC-L (% ^c^)	ID ^d^
α-Pinene	931	1.3	1.3	AI, MS, Co-GC
β-Pinene	973	0.2	0.4	AI, MS, Co-GC
Octen-3-ol	983	0.2	0.8	AI, MS
β-Myrcene	992	0.1	1.3	AI, MS, Co-GC
α-terpinene	931	0.9	1.0	AI, MS, Co-GC
*p*-Cymene	1024	4.4	4.56	AI, MS, Co-GC
γ-terpinene	1055	2.8	3.94	AI, MS, Co-GC
*cis*-Sabinenehydrate	1067	0.4	0.2	AI, MS
Linalool	1101	0.9	0.5	AI, MS, Co-GC
Borneol	1164	0.9	0.7	AI, MS, Co-GC
4-carvomenthenol	1185	0.7	0.5	AI, MS, Co-GC
o-cymen-5-ol	1280	0.2	0.2	AI, MS, Co-GC
2-isopropyl-5-methyl-phenol	1295	0.3	0.2	AI, MS, Co-GC
Carvacrol	1304	77.7	72.8	AI, MS
5-isopropyl-2-methyl phenol	1358	nd	0.3	AI, MS
2-isopropyl-5-methyl-phenyl acetate	1377	0.1	nd	AI, MS
Caryophyllene	1419	1.95	2.2	AI, MS, Co-GC
Spathulenol	1578	0.1	0.2	AI, MS
Carryophyllene oxide	1583	0.5	0.5	AI, MS, Co-GC
Total		93.65	91.6	

^a^ Compounds listed in order of elution from an HP-5 MS capillary column; ^b^ AI: arithmetic indices as determined on an HP-5 MS capillary column using a homologous series of n-alkanes (C9-C23); ^c^ percentage (*w*/*w*) of the identified compound in the essential oil; and ^d^ identification method: AI = arithmetic index, MS = mass spectrum, Co-GC = co-injection with authentic compound, and nd = not detected.

**Table 4:** Composition of the essential oils of *S. montana*.

Compounds ^a^	AI ^b^	SM-B (% ^c^)	SM-D (% ^c^)	ID ^d^
α-Thujene	926	1.3	1.4	AI, MS
α-Pinene	931	0.9	0.7	AI, MS, Co-GC
Camphene	945	0.1	0.8	AI, MS
β-Pinene	973	0.1	0.1	AI, MS, Co-GC
Octen-3-ol	983	nd	0.2	AI, MS
β-Myrcene	992	1.4	1.0	AI, MS, Co-GC
α-Phellandrene	1003	1.03	0.9	AI, MS
δ-2-Carene	1008	0.2	0.3	AI, MS
δ-3-Carene	1015	1.3	1.4	AI, MS, Co-GC
*p*-Cymene	1024	8.9	11.8	AI, MS, Co-GC
Limonene	1027	0.6	0.9	AI, MS
Eucalyptol	1029	0.3	0.4	AI, MS
trans-Ocimene	1040	0.3	0.8	AI, MS
cis-Ocimene	1050	0.13	0.2	AI, MS
γ-Terpinene	1059	4.7	5.4	AI, MS, Co-GC
*cis*-Sabinenehydrate	1067	1.5	4.2	AI, MS
Terpinolene	1087	0.2	0.3	AI, MS
*trans*-Sabinenehydrate	1098	1.4	0.1	AI, MS
Linalool	1101	3.3	0.5	AI, MS, Co-GC
α-Thujone	1104	0.94	0.1	AI, MS
β-Thujone	1116	0.04	tr	AI, MS
*cis*-*p*-Menth-2-en-1-ol	1122	0.2	tr	AI, MS
Camphor	1143	0.3	0.3	AI, MS
Borneol	1164	2.4	2.8	AI, MS, Co-GC
δ-Terpineol	1169	nd	0.7	AI, MS
Terpinene-4-ol	1176	1.98	3.2	AI, MS, Co-GC
*p*-Cymen-8-ol	1187	0.3	0.1	AI, MS
α-Terpineol	1191	0.04	0.2	AI, MS
Thymol methyl ether	1236	1.98	0.1	AI, MS
Carvacrol methyl ether	1244	5.2	5.5	AI, MS
Bornyl acetate	1286	0.04	nd	AI, MS, Co-GC
Thymol	1294	52.8	28.5	AI, MS, Co-GC
Carvacrol	1304	2.5	1.2	AI, MS
Thymyl acetate	1356	0.45	0.5	AI, MS
α-Copaene	1375	0.1	0.1	AI, MS
β-Burbonene	1384	0.1	0.2	AI, MS
β-Caryophyllene	1419	nd	2.3	AI, MS, Co-GC
β-Copaene	1428	nd	0.2	AI, MS
γ-Elemene	1434	nd	0.6	AI, MS
Aromadendrene	1438	nd	0.5	AI, MS
Myltayl-4(12)-ene	1443	nd	nd	AI, MS
α-Carryophyllene	1453	0.6	0.2	AI, MS, Co-GC
Allo-Aromadendrene	1460	0.7	0.2	AI, MS
Dauca-5,8-diene	1474	nd	0.55	AI, MS
γ-Muurolene	1477	nd	0.25	AI, MS
Spathulenol	1578	nd	0.13	AI, MS
Carryophyllene oxide	1583	nd	1.0	AI, MS, Co-GC
Total		98.33	80.83	

^a^ Compounds listed in order of elution from an HP-5 MS capillary column; ^b^ AI: arithmetic indices as determined on an HP-5 MS capillary column using a homologous series of n-alkanes (C9-C23); ^c^ percentage (*w*/*w*) of the identified compound in the essential oil; and ^d^ identification method: AI = arithmetic index, MS = mass spectrum, Co-GC = co-injection with authentic compound, and nd = not detected. Concentrations below 0.05% are marked as tr (traces).

**Table 5:** Antioxidant activity of EOs expressed as IC_50_ values (μg/mL).

Species	EOs from Samples	DPPH μg/mL	ABTS μg/mL
*Origanum* *vulgare* subsp. *hirtum*	OV-L	530 ± 8	110 ± 10
	OV-P	600 ± 12	120 ± 8
*Thymbra* *capitata*	TC-M	530 ± 9	180 ± 9
	TC-L	570 ± 8	220 ±13
*Satureja* *montana*	SM-B	1200 ± 5	460 ± 27
	SM-D	820 ± 4	500 ± 14

**Table 6:** Minimum Inhibitory Concentration of essential oils against six pathogens.

Species	EO from Sample	MIC (mg/mL)					
		*E. coli* ATCC 10535	*S.* Enteritidis ATCC 49223	*P. aeruginosa* ATCC 9027	*M. luteus* ATCC 10240	*S. maltophilia* ATCC 13637	*C. albicans* ATCC 10231
*Origanum* *vulgare* subsp. *hirtum*	OV-L	0.312	1.250	1.250	0.312	0.156	0.312
	OV-P	0.625	0.625	1.250	0.625	0.156	0.312
*Thymbra* *capitata*	TC-M	0.312	0.625	2.5	0.625	0.156	0.156
	TC-L	0.625	1.250	NO MIC	0.625	0.312	0.156
*Satureja* *montana*	SM-B	0.625	1.250	NO MIC	0.625	1.250	0.312
	SM-D	1.250	2.5	2.5	0.625	0.625	0.625

**Table 7:** Comparison of yield, chemical composition, and biological activity of the essential oils from plants taken in study.

Species	EO from Sample	Yield (% ^a^)	Key Active Components	Antioxidant Activity (DPPH/ABTS, μg/mL ^b^)	Antimicrobial Spectrum ^c^
*Origanum vulgare* subsp. *hirtum*	OV-L	4.06	Carvacrol (74.6%), γ-Terpinene	530/110	Broad, strong
	OV-P	3.83	Carvacrol (79.8%), γ-Terpinene	600/120	Broad, strong
*Thymbra capitata*	TC-M	1.66	Carvacrol (77.7%), *p*-cymene	530/180	Broad, strong
	TC-L	0.75	Carvacrol (72.8%), *p*-cymene	570/220	Moderate, inactive against *P. aeruginosa*
*Satureja montana*	SM-B	0.39	Thymol (52.8%), *p*-Cymene	1200/460	Weak, inactive against *P. aeruginosa*
	SM-D	0.73	Thymol (28.5%), *p*-Cymene	820/500	Weakest overall

^a^ mL of EO per 100 g of dried plant material; ^b^ expressed as IC_50_ values; ^c^ tested against four Gram-negative and one Gram-positive bacteria and one fungus.

**Table 8:** Essential oil nanoemulsions characterizations.

Nanoemulsion of EO from Sample	Particle Size (nm ^a^)	PDI	Z-Potential (mV ^b^)
OV-L	132.4 ±15.3	0.152 ±0.126	−11.2 ± 2.8
TC-M	191.8± 17.2	0.076 ±0.09	−9.6 ± 0.5

^a^ nm—nano meters; ^b^ mV—millivolts.

**Table 9:** Plant samples.

Nr.	Species	Location	Plant Code	Collection Date	Altitude * (m.a.s.l.)	Coordinates	
						Latitude (N)	Longitude (E)
1.	*Origanum vulgare* subsp. *hirtum*	Lukovë	OV-L	7 August 2023	~55	39°97′88″	19°91′29″
2.		Qafë Pishë	OV-P	4 August 2023	~1200	40°25′92″	19°79′11″
3.	*Thymbra* *capitata*	Mallakastër	TC-M	4 August 2023	~54	41°58′47″	20°13′12″
4.		Lukovë	TC-L	13 August 2023	~179	39°98′19″	19°91′71″
5.	*Satureja* *montana*	Bego Mauntain	SM-B	4 August 2023	~1500	40°25′44″	19°78′86″
6.		Dajti Mountain	SM-D	19 August 2023	~724	41°36′90″	19°94′20″

* m.a.s.l.—meters above sea level.

## Data Availability

The original contributions presented in the study are included in the article and Supplementary Materials; further inquiries can be directed to the corresponding authors.

## Supplementary Materials

The following supporting information can be downloaded at: https://www.mdpi.com/article/10.3390/molecules30163329/s1, Figure S1: GC-MS chromatogram of *O. vulgare* subsp. *hirtum* EO—Sample OV-L; Figure S2: GC-MS chromatogram of *O. vulgare* subsp. *hirtum* EO—Sample OV-P; Figure S3. GC-MS chromatogram of *Thymbra capitata* EO—Sample TC-M; Figure S4. GC-MS chromatogram of *Thymbra capitata* EO—Sample TC-L; Figure S5. GC-MS chromatogram of *Satureja montana* EO—Sample SM-B; Figure S6. GC-MS chromatogram of *Satureja montana* EO—Sample SM-D.
