# Supplementary material for: Essential Oils from Wild Albanian Lamiaceae: GC-MS Profiling, Biological Activity, and Enhanced Delivery via Nanoencapsulation
Source: Molecules. 2025 Aug 9;30(16):3329. doi: 10.3390/molecules30163329 (PMC12388121; doi:10.3390/molecules30163329)
Supplement: Supplementary file 1 [file molecules-30-03329-s001.zip › molecules-3783783-supplementary.pdf]

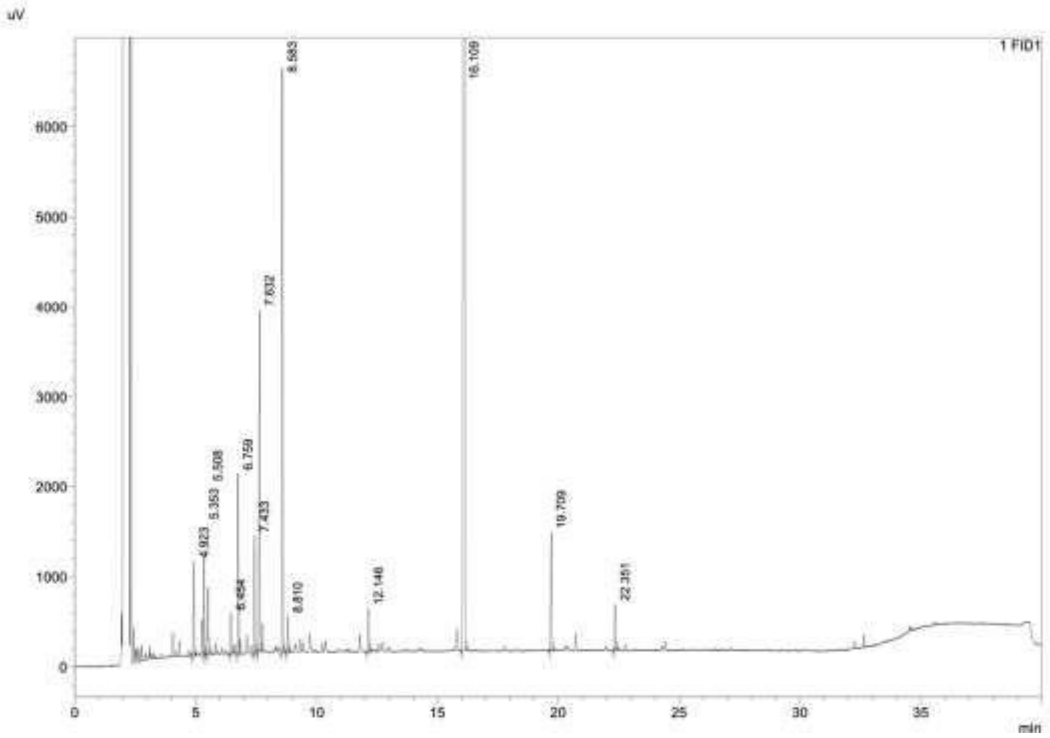

Figure S1. GC-MS chromatogram of *O. vulgare* subsp. *hirtum* EO - Sample OV-L

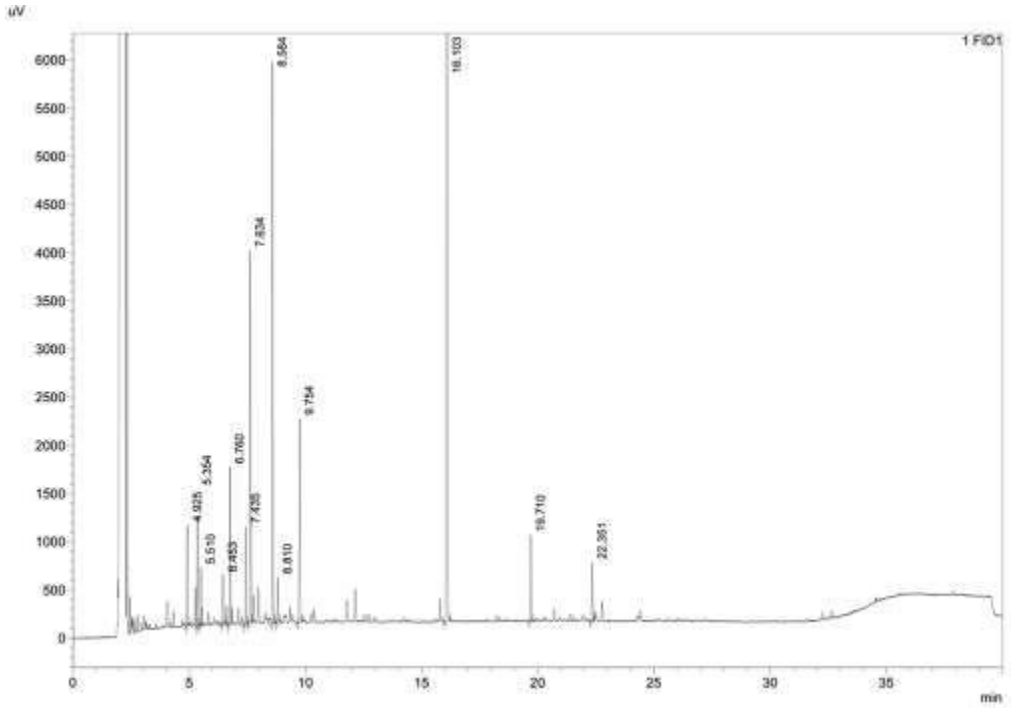

Figure S2. GC-MS chromatogram of *O. vulgare* subsp. *hirtum* EO - Sample OV-P

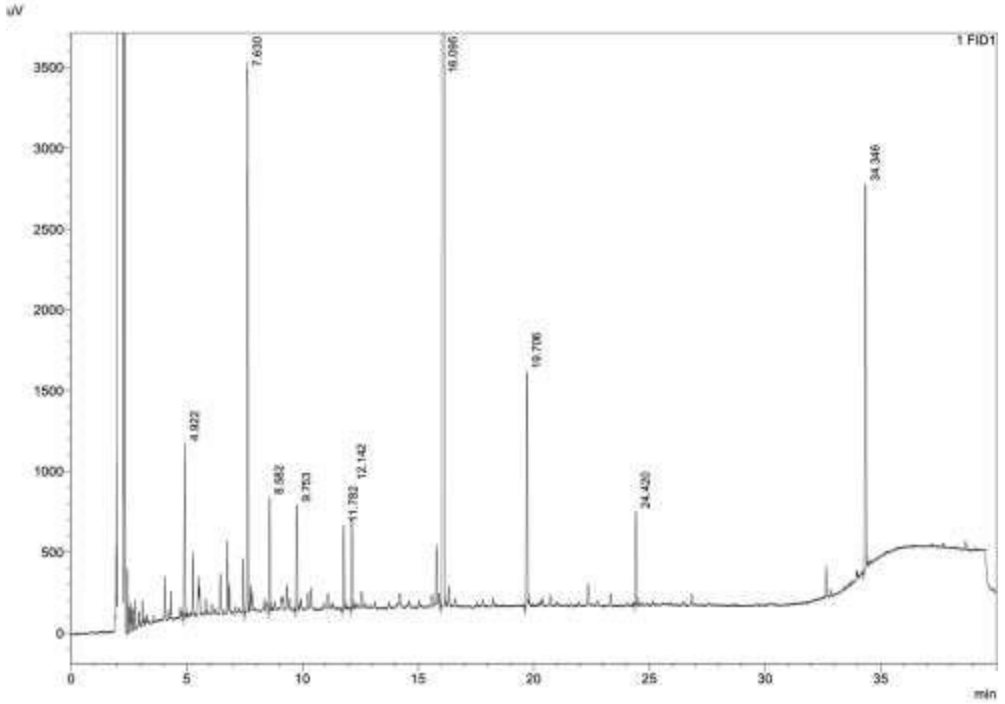

Figure S3. GC-MS chromatogram of *Thymbra capitata* EO - Sample TC-M

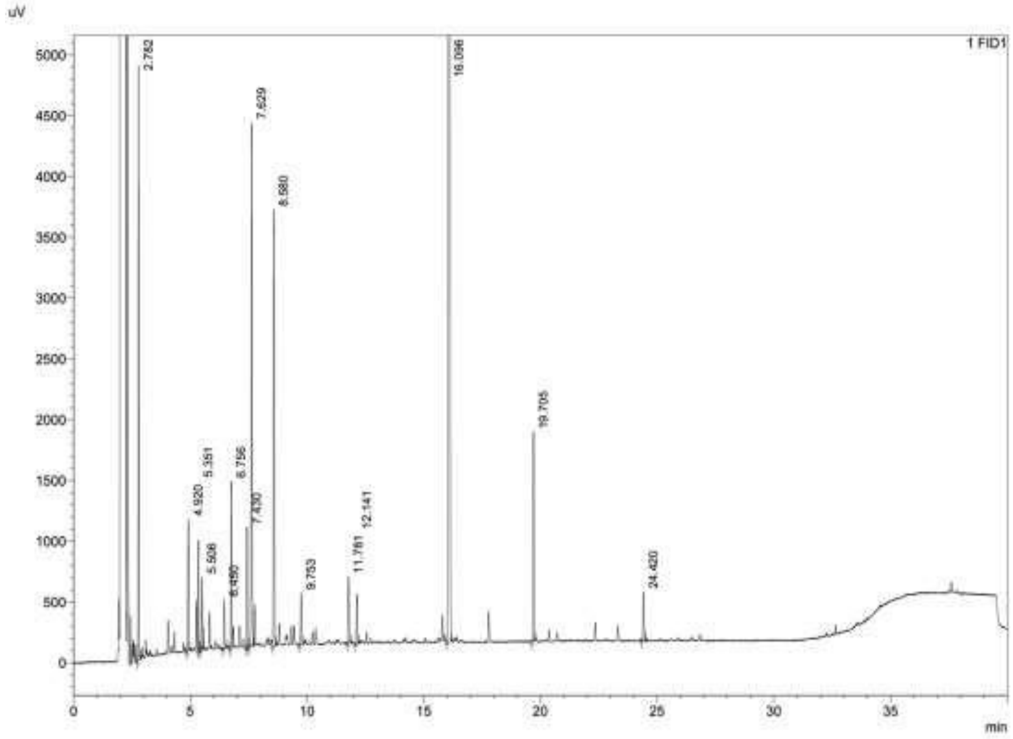

Figure S4. GC-MS chromatogram of *Thymbra capitata* EO - Sample TC-L

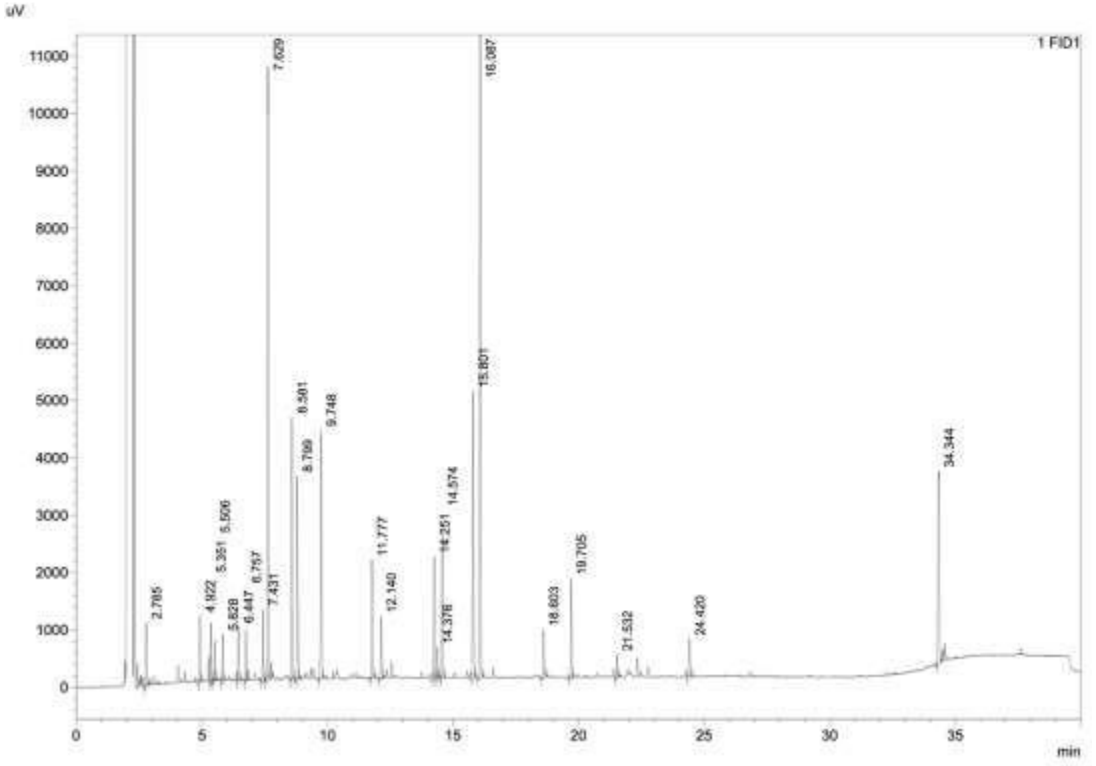

Figure S5. GC-MS chromatogram of *Satureja montana* EO - Sample SM-B

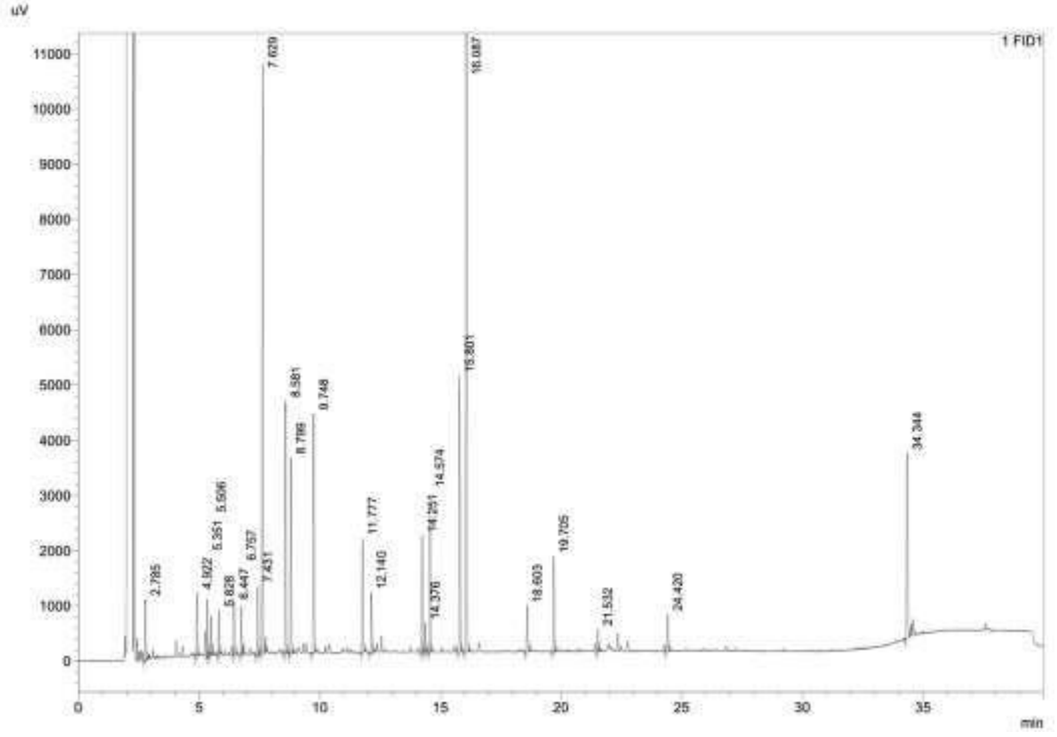

Figure S6. GC-MS chromatogram of *Satureja montana* EO - Sample SM-D
